# Impact of episiotomy on pelvic floor disorders and their influence on women's wellness after the sixth month postpartum: a retrospective study

**DOI:** 10.1186/1472-6874-11-12

**Published:** 2011-04-18

**Authors:** Serena Bertozzi, Ambrogio P Londero, Arrigo Fruscalzo, Lorenza Driul, Cristina Delneri, Angelo Calcagno, Paolo Di Benedetto, Diego Marchesoni

**Affiliations:** 1 Clinic of Surgical Semeiotics, AOU "SM della Misericordia" of Udine, 33100 Udine Italy; 2 Clinic of Obstetrics and Gynecology, AOU "SM della Misericordia" of Udine, 33100 Udine Italy; 3 Frauenklinik, Mathias-Spital, Frankenburgstr. 31, 48431 Rheine, Germany; 4 Istituto di Medicina Fisica e Riabilitazione Gervasutta, Dipartimento di Medicina Riabilitativa, servizo di Diagnosi e Riabilitazione Perineale, 33100 Udine, Italy

**Keywords:** episiotomy, vaginal delivery, pelvic floor disorders, perineum, psycho-physical health

## Abstract

**Background:**

The role of episiotomy as a protective factor against pelvic floor disorders postpartum has been debated for many years, but its routine use has been hitherto discouraged in the literature. Comparisons between restrictive and routine use of episiotomy in existent literature, however, fail to include any consideration relating to quality of life. The aim of this study, therefore, is to state the role of episiotomy in preserving the perineum from damage, in order to prevent the influence of pelvic floor disorders on women's psycho-physical wellness after the sixth month postpartum.

**Methods:**

A follow-up telephone interview was performed among 377 primiparous and secondiparous Caucasian women who had a child by spontaneous or operative vaginal delivery in 2006 using a self-created questionnaire and King's Health Questionnaire (KHQ).

**Results:**

The mean age at delivery was 35.26 (±4.68) years and episiotomy was performed in 59.2% of women. Multivariate linear regression shows episiotomy associated to higher quality of life after the sixth month postpartum by correlating with inferior values of King's Health Questionnaire (p < 0.05).

**Conclusions:**

Episiotomy appears to be a protective factor for women's wellness. Women who had episiotomy and who experienced perineal symptoms have a better psycho-physical health status in the 12.79 months (±3.3) follow-up.

## Background

Providing assistance in cases of spontaneous vaginal delivery presents a valuable opportunity to prevent perineal disorders such as urinary incontinence (UI), which, requiring surgical intervention in circa 400,000 women every year in the USA alone, has been compared to a hidden epidemic [1].

UI prevalence rate in women is estimated at between 10% and 50% depending on age [2-5] - a study involving 1029 women with a mean age of 53 years in our region found a UI prevalence of around 44% [6]. UI in women is often assumed to be attributable to the effects of pregnancy and childbirth. In fact, among pregnant women, UI is a common occurrence compared with other groups of women, with reported prevalence rates ranging between 31% and 60% [7,8]. However, UI tends to be a self-limited condition postpartum, with persistent postpartum UI prevalence rates cited as variating between 0.7% and 44% [9-11].

In addition to UI, many perineal disorders are commonly associated with vaginal delivery, such as anal incontinence, chronic pelvic pain, other lower urinary tract symptoms and dyspareunia, incidences of which are probably underestimated.

Recent studies underline the importance of a better delivery management [12,13] in order to prevent perineal damage caused by vaginal delivery and its complications, represented by mother-newborn bonding failure in the short-term and pelvic floor disorders at the long-term follow up.

Episiotomy itself remains controversial since its first use by a Scottish midwife in the 1740s [14]. At the beginning of the 1980s, episiotomy was widely performed, despite no clear demonstration of its efficacy [15]. During the 1980s and the 1990s, episiotomy was found to have more side effects than benefits: increased blood loss, greater postpartum pain and dyspareunia, more difficult and lengthy repair, higher incidence of third to fourth degree tears, and no evident protection of foetal health [16,17]. Therefore, opinion has shifted, with restrictive episiotomy policies appearing to have a number of benefits as reported in the most recent Cochrane review [18].

Some authors found an association between episiotomy and more perineal pain and dyspareunia during the early postpartum weeks, even when compared with cases presenting spontaneous tears [19-21]. Perineal trauma during delivery results in perineal pain regardless of whether episiotomy was performed or not, and independent of presence of tear and the methods used to repair it [22,23]. Fortunately, painless intercourse is generally resumed by 6 weeks postpartum, and the effects of delivery trauma on sexual function generally disappear by 12 months [24].

In the literature, there are no studies that evaluate the effect of episiotomy on women's quality of life in relation to lower urinary tract symptoms postpartum - most of the studies simply evaluate the presence or absence of such symptoms, commonly urinary incontinence.

In the present study we analyse the maternal, neonatal, and obstetric factors influencing the quality of life of women who vaginally delivered, evaluated by the King's Health Questionnaire (KHQ) at the 12.79 months (±3.3) postpartum follow-up, and focusing in particular on the role of episiotomy. We investigate both physical and psychological health status, and their corresponding effects on daily life, basing our consideration on the most recent definition of health by the World Health Organisation as the coexistence of physical, psychological and social wellness.

## Methods

From the women who gave birth in our clinic during 2006, 900 consecutive Caucasian primiparous and secondiparous women were selected for interview. The population included both vaginal deliveries and caesarean sections. Data was collected for a study of prevalence of lower urinary tract and perineal symptoms, including sexual function, and quality of life postpartum [25,26]. The final population, after data collection, totalled 602 women with a 66.9% response rate (602/900) including caesarean sections [26]. For the purposes of this study, the group of 377 women who delivered vaginally was of relevance and was therefore included. Exclusion criteria were parity ≥2, prematurity, multiple pregnancies, lack of ultrasonographic confirmation of the gestational age within the 20th gestational week, non-Caucasian women, and caesarean section. This study was conducted according to the declaration of Helsinki, and after internal review board approval.

The names, addresses and telephone numbers of the 900 consecutive Caucasian primiparous and secondiparous women who formed our population were gathered via interrogation of the Clinic's digital information system. A single operator subsequently contacted the women by telephone. Clinical data relating to hospitalisation period and obstetric and neonatal outcome were consequently collected using paper files held by the Clinic.

The women were asked to complete a self-created questionnaire (29 questions) and the validated Italian version of the KHQ [27]. In specific, the first questionnaire investigates the presence of the following symptoms at the time of the interview: urinary stress; urge and mixed incontinence; increased daytime voiding frequency and urgency; nocturia; voiding symptoms; feeling of incomplete bladder emptying; dyspareunia; chronic pelvic discomfort; faecal incontinence; and recurrent urogenital infections, together with the timing of such disorders in relation to pregnancy.

The KHQ is a condition-specific preference-based measure of health that was originally designed to assess the quality of life of women with UI and LUTS [27]. Its twenty-one items cover eight dimensions of health: urinary symptoms severity; role limitations; physical functioning; social functioning; emotional problems; personal relationships; sleep disturbance; and general health. Higher scores of KHQ indicate greater impairment in quality of life. For the purposes of this study, the following symptoms are classified as lower urinary tract symptoms (LUTS): UI; urgency; nocturia; increased daytime frequency; voiding symptoms; feeling of incomplete emptying; perineal pain; pelvic pain; bladder pain; dyspareunia.

We defined perineal dysfunctions as permanent when affecting women after the sixth month postpartum; urinary incontinence (UI) and definitions of other pelvic floor symptoms are based on the last International Continence Society standardisation publication [28].

The following factors were included in our consideration: maternal age; pre-gestational BMI; BMI at term; weight gain during pregnancy; tobacco smoking during pregnancy; constipation; duration of the I and II stages of labour; Kristeller manoeuvre (fundal pressure during the II labour stage to accelerate the foetal expulsion); epidural analgesia; type of delivery (spontaneous or operative); previous surgery (including laparoscopic and laparotomic interventions in the lower quadrants of the abdomen, transvaginal surgery, isteroscopy, and uterine curettage); neonatal age; weight and length at birth; previous UI; permanent UI; dyspareunia; presence of tears (first, second, or third to forth degree perineal-vaginal tears); and whether or not episiotomy was performed.

In our Clinic, episiotomy is always performed mediolaterally. Under normal circumstances, the incision measures a total length of 4 cm and a depth of 3 cm, and is positioned, at an angle of 45°, on the right side of the vulva, within 1 cm of the posterior commissure of the vaginal orifice. This initial incision is never extended, and episiotomy is performed when the tissues are stretched by the baby's head. Moreover episiotomy is always performed when operative vaginal delivery is carried out - vacuum extraction being the sole choice of method for such interventions in our clinic.

Interrupted sutures are used to repair the perineal muscles. To close the skin, interrupted Donati stitch or subcuticular stitching is used, whilst the vagina is repaired using either continuous or interrupted sutures, the method employed in both the latter being at the discretion of the individual suture operator. In our University Clinic, due to a generally uniform approach to suturing, the skin is invariably closed using interrupted Donati stitching and the vagina by continuous suture. In all of the analysed cases, polyglactin suture was used for short-term wound support (7 to 10 days).

The collected data was analysed using bivariate and multivariate linear regression. In addition, bivariate analyses were carried out in order to evaluate the statistical association between variable pairs, with the KHQ score as the dependent variable. The independent or explanatory variables were either continuous (returning a fixed value) or dichotomous (returning either a positive or zero value depending on whether the respondent reported presence or absence of a specific trait).

Each linear regression model was assigned a coefficient and a 95% confidence interval, as calculated by means of an F test for the null hypothesis that the slope of the regression line is zero. A multivariate linear regression was, additionally, performed for the most relevant parameters. In order to test the continuous variables, we performed a t-test, and the Wilcoxon test where appropriate. Statistical significance was defined as p < 0.05. Statistical evaluations were performed using R (a language and environment for statistical computing) Version 2.10.1.

## Results

The mean age at delivery of women in the data set was calculated as 35.26 years (±4.68), the mean BMI before pregnancy 22.04 kg/m^2 ^(±3.74) with a comparable value at the interview stage, and the mean weight gain during pregnancy was 13.19 kg (±4.45) [Table 1]. The mean KHQ score was 136.73 (±101.43). 39.5% of cases had undergone surgery previously, with 4.0% previously undergoing caesarean section. 19.6% of women smoked and 19.3% reported constipation [Table 1].

**Table 1:** Population characteristics.

Maternal characteristics	
Maternal age (years)	35.26 (±4.68)

Secondiparous women	45.4% (171/377)*

BMI before pregnancy (Kg/m^2^)	22.04 (±3.74)

Weight gain during pregnancy (Kg)	13.19 (±4.45)

Actual BMI (Kg/m^2^)	22.05 (±3.76)

Previous surgery	39.5% (148/375)*

N° of previous surgical interventions	0 (0 - 5)**

Previous cesarean section	4.0% (15/373)*

Number of previous cesarean sections	1 (1 - 2)**

Tobacco smoke	19.6% (73/373)*

Constipation	19.3% (72/373)*

**Labour and delivery characteristics**	

Gestational age at birth (weeks)	39.68 (±1.34)

First labour stage (min)	256.05 (±222.08)

Second labour stage (min)	38.21 (±33.38)

Epidural analgesia	18.2% (58/319)*

Spontaneous delivery	83.8% (316/377)*

Operative delivery	16.2% (61/377)*

Intact perineum	6.6% (25/377)*

First degree vagino-perineal tears	20.4% (77/377)*

Second degree vagino-perineal tears	15.9% (60/377)*

Third-fourth degree vagino-perineal tears	1.3% (5/377)*

Episiotomy	59.4% (224/377)*

Neonatal weight (g)	3364 (±419)

Neonatal lenght (cm)	50.95 (±2.27)

Data are given as: mean (± SD), * Percentage and absolute values, ** Median (range).

The mean gestational age at delivery was 39 weeks and 4 days (±8 days), mean neonatal weight at birth 3364 gr. (±419) and length 50.95 cm (±2.27). Vaginal delivery was spontaneous in 83.8% of cases and operative in the remainder. Epidural analgesia was performed in 18.2% of cases. Episiotomy was required in 59.4% of cases; 6.6% of women had an intact perineum. Tears were present to the first degree in 20.4% of cases, second degree in 15.9%, and third to fourth degree in 1.3% [table 1].

All cases of third to fourth degree tears and operative delivery are included in the episiotomy subgroup with a prevalence respectively of 2.2% (p 0.063) and 26.3% (p < 0.05). Moreover, there is a lower proportion (29.5%) of secondiparous women in the episiotomy subgroup than in the subgroup of women who did not receive an episiotomy (68.6%) (p < 0.05).

Dyspareunia was reported by 17.2% of women. Among the group of vaginal deliveries, SUI had a prevalence rate of 27.6%, UUI 14.8%, MUI 11.9%, and LUTS 41.4%. Among the women who received an episiotomy, 30.0% were secondiparous - significantly lower than women in the same group with an intact perineum, first degree tears, or second degree tears (68.0%, 64.0%, and 75.0% respectively) (p < 0.05). 21.0% of the secondiparous women reported previous UI. The prevalence of LUTS postpartum is 30.1% in primiparous women, 25.2% in secondiparous women without previous incontinence, and 94.4% in secondiparous women with previous UI. The prevalence rates in the first and second of these groups are comparable, and both are significantly lower than the third group (p < 0.05).

Table 2 presents the monovariate linear regression analysis of parameters plausibly influencing the KHQ score. Maternal age at delivery, previous UI, third to fourth degree tears, performance of an episiotomy procedure, UI, and dyspareunia significantly influence KHQ score. Higher age, history of previous surgery, previous urinary incontinence, higher neonatal weight, presence of third and fourth degree tears, urinary incontinence and dyspareunia are associated with higher KHQ scores. Epidural analgesia and episiotomy are associated with lower KHQ scores at the follow-up.

**Table 2:** Linear regressions, dependent variable KHQ score

	Coefficent (CI 95%)	p
**Maternal characteristics**		

Maternal age	2.671 (0.484 - 4.857)	<0.05

Actual BMI	1.215 (-1.529 - 3.96)	0.384

BMI before pregnancy	0.809 (-1.959 - 3.576)	0.566

Weight gain during pregnancy	-0.113 (-2.436 - 2.21)	0.924

Tobacco smoke	1.079 (-24.969 - 27.126)	0.935

Constipation	14.742 (-11.377 - 40.862)	0.268

Previous surgery	19.112 (-1.843 - 40.068)	0.074

Parity	15.04 (-5.564 - 35.643)	0.152

Time before follow up	-0.963 (-4.178 - 2.252)	0.556

Previous urinary incontinence	70.252 (37.241 - 103.263)	<0.05

**Labour and delivery characteristics**		

Gestational age	3.846 (-4.611 - 12.303)	0.372

Neonatal weight	0.023 (-0.001 - 0.048)	0.065

Neonatal length	2.854 (-2.865 - 8.573)	0.327

Epidural analgesia	-25.579 (-53.968 - 2.809)	0.077

First labour stage	0.028 (-0.019 - 0.075)	0.241

Second labour stage	-0.201 (-0.51 - 0.107)	0.201

Kristeller manouvre	-8.427 (-33.847 - 16.992)	0.515

Operative delivery	-13.831 (-41.725 - 14.062)	0.330

Episiotomy	-28.964 (-49.702 - -8.226)	<0.05

Perineal-vaginal tears	27.706 (6.857 - 48.555)	<0.05

Second degree tears	-1.782 (-29.898 - 26.333)	0.901

Third and fourth degree tears	90.581 (1.144 - 180.019)	<0.05

**Urinary and pelvic symptoms at follow up**		

Urinary incontinence	125.693 (107.359 - 144.027)	<0.05

Stress urinary incontinence	122.044 (102.653 - 141.435)	<0.05

Urge urinary incontinence	123.418 (97.353 - 149.483)	<0.05

Mixed urinary incontinence	126.94 (97.953 - 155.928)	<0.05

Dyspareunia	29.357 (2.292 - 56.422)	<0.05

LUTS	88.764 (69.926 - 107.602)	<0.05

**Figure 1:**
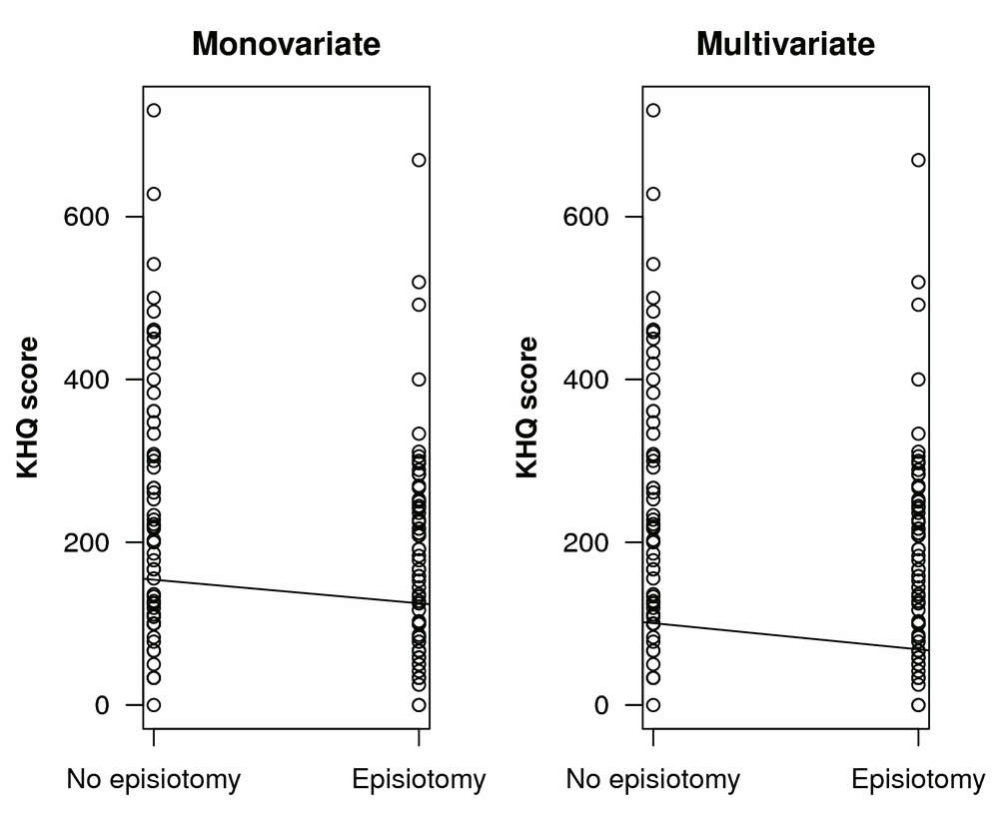
**Monovariate and multivariate linear regressions: dependant variable is KHQ score; independent variable is presence/non-presence of episiotomy surgery**.

Table 3 displays the final model of the multivariate logistic regression with the KHQ score as the dependent variable. The results of this model reaffirm those of the monovariate model; namely that episiotomy and UI independently influence the KHQ score, the former correlating with a higher quality of life (lower KHQ score) [Figure 1] and the latter with a lower one (higher KHQ score). On the other hand, second degree tears, third to fourth degree tears, previous UI, and dyspareunia were not proved to have statistical significance in the multivariate analysis. The correlation between episiotomy and low KHQ score in the multivariate model is independent of previous UI, parity, and time to follow up.

**Table 3:** Multivariate linear regressions, dependent variable KHQ score.

Maternal characteristics	Effect (CI 95%)	p
Maternal age	0.469 (-1.72 - 2.658)	0.674

Previous surgery	13.988 (-5.727 - 33.703)	0.164

Parity	-9.617 (-32.943 - 13.709)	0.418

Time before follow up	-1.478 (-4.392 - 1.435)	0.319

Previous urinary incontinence	25.945 (-9.373 - 61.263)	0.149

**Labour and delivery characteristics**		

Neonatal weight	0.015 (-0.008 - 0.038)	0.190

Epidural analgesia	-22.662 (-51.039 - 5.715)	0.117

Episiotomy	-36.146 (-59.077 - -13.216)	<0.05

Second degree tears	-22.495 (-51.54 - 6.55)	0.129

Third and fourth degree tears	38.257 (-52.405 - 128.919)	0.407

**Urinary and pelvic symptoms at follow up**		

LUTS	81.947 (60.934 - 102.96)	<0.05

The initial model was: maternal age, current BMI, constipation, previous surgery excluded Cesarean sections, parity, time between delivery and telephone interview, neonatal weight at birth, epidural analgesia, I and II labour stage duration, Kristeller manoeuvre, operative delivery, II and III to IV vaginal-perineal tears, episiotomy performance, persistent dyspareunia, previous and actual UI.

**Figure 2:**
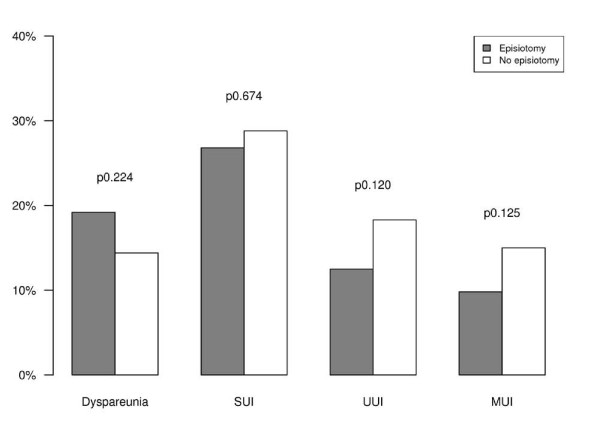
**Prevalence rates of dyspareunia, stress urinary incontinence (SUI), urge urinary incontinence (UUI), mixed urinary incontinence (MUI), low urinary tract symptoms (LUTS) in women with episiotomy and in women without**.

KHQ score results were significantly lower in the subgroup of women who received an episiotomy, with an average score of 124.98 (±81.46) in comparison to the subgroup of those who did not, whose score averaged 153.94 (±123.34). Moreover, although the differences do not measure as statistically significant, it is interesting to note that the episiotomy subgroup presented lower prevalence rates of UI, SUI, UUI, and MUI. In addition, the episiotomy subgroup did not demonstrate a significantly higher prevalence of dyspareunia, or LUTS in general [Figure 2].

Table 4 describes in detail the health aspects considered by the KHQ in patients with LUTS after delivery. Our results conclude that episiotomy causes a reduction in the impact of LUTS on the following dimensions of health: urinary symptoms severity; role limitations; physical limitations; social limitations; and personal relationships (p < 0.05).

**Table 4:** Differences between subgroup with episiotomy and the subgroup without in patiens who suffers LUTS at the time of follow up.

	No episiotomy	Episiotomy	p
KHQ score	252.07 (±164.93)	178.52 (±121.19)	<0.05

General health perceptions	40.61 (±27.74)	40.89 (±27.17)	0.954

Urinary symptoms severity	50.91 (±31.33)	36 (±27.27)	<0.05

Role limitations	33.33 (0-83.33)	0 (0-100)	<0.05*

Physical limitations	33.33 (0-100)	0 (0-83.33)	<0.05*

Social limitations	11.11 (0-100)	0 (0-88.89)	0.097*

Personal relationships	78.03 (±29.72)	53.62 (±26.09)	<0.05

Emotions	0 (0-33.33)	0 (0-44.44)	0.177*

Sleep/energy	0 (0-100)	0 (0-100)	0.458*

Severity measures	8.33 (0-58.33)	8.33 (0-66.67)	0.874*

Data are given as mean (± SD) (p - t-test) or where appropiate as median (range) (p - Wilcoxon test) (*).

## Discussion

Pelvic floor disorders are a highly common condition in the immediate postpartum: their prevalence is estimated at about 18.4% in primiparous women and 24.6% in secondiparas [29]. They tend to remit after the sixth month following delivery, although in a considerable number of cases they do persist, and the vaginal delivery management appears to offer an opportunity to reduce their morbidity [1]. Amongst our population, pelvic floor disorders are present at around one year postpartum in 40% of all cases, and have a substantial effect on the psycho-physical health of women; yet the majority of cases remain untreated and in reality only 4.49% of our population has been referred to a pelvi-perineal rehabilitation treatment [25].

Our study provides evidence that episiotomy is associated with significantly lower KHQ scores at the time of follow-up. As shown in table 4 the KHQ score for patients with LUTS is significantly lower when an episiotomy is performed (p < 0.05).

The data in our study, as in the literature, suggests that some women are predisposed to develop pelvic floor disorders during pregnancy and postpartum [30]. In specific, secondiparous women with previous UI are more likely to develop LUTS after the second delivery than secondiparous women without previous UI. In addition to the maternal predisposing factors, many obstetric factors play a part in determining the severity of perineal damage where it continues to affect the quality of life at a 12.79 month (±3.3) postpartum follow-up: in particular, the presence of third to fourth degree tears at delivery, and the onset of UI and dyspareunia.

Likewise, it has been proposed that certain factors offer protection against pelvic floor disorders. In particular, the role of episiotomy is widely debated, but its routine use has been thus far discouraged. In our opinion, and in contrast to the current literature, episiotomy could serve as a protective factor for pelvic floor disorders when considered in terms of quality of life, since women who received an episiotomy and experienced perineal symptoms in the early postpartum have a better psycho-physical health status at the mid-term follow-up.

In a recent Cochrane Collaboration review, which, analysing a population of 5541 women, compared restrictive use of episiotomy with routine use during vaginal birth, a restrictive episiotomy policy results in less severe perineal trauma, less suturing, fewer healing complications but also more anterior perineal trauma. The same review notes no differences between policy in relation to urinary incontinence or pain measures [18]. The majority of studies that we analysed carried out a limited follow-up and failed entirely to evaluate quality of life. It is our considered opinion that both factors form a key part of any investigation that seeks to assess the impact of perineal trauma, to clarify the role of episiotomy in relation to pelvic function disorders, and to evaluate the consequences of such symptoms on women's quality of life.

In addition to the above, there is a strong case that the safety and efficacy of episiotomy is related closely to the methods employed in performing the surgery. Midline episiotomy allows for a better wound healing with an improved appearance of the scar and a better future sexual function. On the other hand, it risks being extended backwards, causing anal sphincter injury, additional risk factors for which are instrumental assistance, prolonged II labour stage and occipito-posterior position [31]. In order to reduce the risk of severe perineal trauma, some authors recommend mediolateral episiotomy in order that any extension to the incision does not lacerate the anal sphincter [32].

In our study, the results of a multivariate linear regression demonstrate that episiotomy is an independent protective factor against higher KHQ scores; in fact, the mean KHQ score is significantly lower in the episiotomy subgroup than in the subgroup with no episiotomy. The differences in KHQ score in patients complaining of LUTS relate mainly to the categories of urinary symptoms severity, personal relationships, the role and physical limitations (p < 0.05), and also social limitations. These results suggest that pelvic symptoms exert a notable influence on quality of life in the subgroup of women who did not receive an episiotomy.

Whilst not statistically significant, it is interesting to note that Figure 2 shows a lower incidence of SUI, UUI, and MUI in the episiotomy subgroup. Symptoms of urge are, in particular, notably more prevalent amongst women who did not receive episiotomy (p n.s.), which could be due to the higher rate of perineal trauma observed in the restrictive episiotomy policies [18]. Although this cannot be proved statistically, we are of the belief that the higher likelihood of such trauma could be an explanation for the higher KHQ score at the mid-term follow-up for women who did not receive an episiotomy and who suffer from LUTS.

Postpartum dyspareunia was found to be unrelated to episiotomy at the mid-term follow-up [26], and Figure 2 shows the rate of dyspareunia in the episiotomy subgroup as being not significantly higher than that of the subgroup with no episiotomy.

The weakness of our study is its retrospective organisation. One of the biases to consider is the inconsistency in the time between delivery and our follow-up - all phone calls were carried out during a short period of time, meaning follow-up times fluctuated greatly between individuals. In reality, an assessment of this bias by multivariate logistic regression analysis concludes that it has no influence on results. The strengths of our study lie in its methodology, employing a global psycho-physical investigation to evaluate women's health, and assessing results at the mid to long term follow-up.

## Conclusions

On the basis of our results, we propose that, when carrying out randomised clinical trials to compare routine versus conservative episiotomy policies, any conclusions should be considered in the context of quality of health. Moving forward, it is important to standardise the classification of restrictive episiotomy policy. Defining precise indications would overcome the varying interpretations which are currently evident, where rates of episiotomy in conservative policy groups can range from as low as 7.6% up to 80% [18]. A more complete understanding of the factors leading to perineal damage during delivery would enable the definition of a higher risk population, thus allowing a meaningful classification to be proposed. Finally, a point of key importance is standardisation of episiotomy techniques. Such standards would not only enable comparisons between studies, but also maintain consistency between delivery managements.

In conclusion, our study reports an association between episiotomy and a low KHQ score, showing that those women who received an episiotomy and who present LUTS at the 12.79 months (±3.3) follow-up postpartum have a higher quality of life.

## Abbreviations

KHQ: King's Health Questionnaire; UI: Urinary Incontinence; SUI: Stress Urinary Incontinence; UUI: Urge Urinary Incontinence; MUI: Mixed Urinary Incontinence; LUTS: Low Urinary Tract Symptoms.

## Competing interests

The authors declare that they have no competing interests.

## Authors' contributions

APL, SB, and CD planned the study and wrote the protocol. SB, APL collected the data. SB, AF, LD, AC carried out literature research and drafted the manuscript. APL, SB, AF, LD analyzed the data and drafted the manuscript. CD, PDB, and DM helped in drafting and the critical revision of the manuscript. All authors read and approved the final manuscript.

## Pre-publication history

The pre-publication history for this paper can be accessed here:

http://www.biomedcentral.com/1472-6874/11/12/prepub
